# Complication following primary repair of a penetrating bull horn injury to the trachea

**DOI:** 10.4103/0974-2700.43199

**Published:** 2008

**Authors:** Mozaffar M Khan, Syed Moied Ahmed, Mohd. Shakeel, Adil Hasan, Sarvesh Pal Singh, Masood M Siddiqi

**Affiliations:** 1 Department of Anesthesiology and Critical Care, JN Medical College, AMU, Aligarh, India; 1 Department of Otorhinolaryngology, JN Medical College, AMU, Aligarh, India

**Keywords:** Elective controlled ventilation, penetrating tracheal injury, subcutaneous emphysema

## Abstract

A 22-year-old male patient was admitted to the casualty with a bull horn injury in the lower zone of the neck in the midline. The patient was conscious and distressed but hemodynamically stable. Local examination revealed a lacerated wound. He underwent emergency primary repair of the wound under halothane anesthesia; intubation was done keeping in readiness all preparations for difficult airway management. Postoperatively, elective controlled ventilation was performed with continuous infusion of muscle relaxant. After approximately 8 hours of controlled ventilation, the syringe pump failed; this initially went unnoticed and made the patient cough and buck on the tube. Infusion was restarted after a bolus dose of vecuronium bromide intravenously but, meanwhile, the patient developed subcutaneous emphysema in the neck. He was immediately transferred to the operating room, where exploration of the surgical site revealed dehiscence of the tracheal wound; this had led to the subcutaneous emphysema. Repair of the tracheal wound dehiscence was not possible due to both lack of space and lack of tissue for apposition. Hence, a tracheostomy tube was inserted through the tracheal wound and the patient was transferred to the intensive care unit for elective controlled ventilation. The patient was weaned off the ventilator within 24 h and transferred to the surgical ward on spontaneous ventilation with the tracheostomy tube in situ. The size of the patient's tracheostomy tube was reduced gradually by the serial exchange method. The wound ultimately healed with minimal scarring.

## INTRODUCTION

Upper airway injury with blunt and penetrating trauma is rare but life threatening and 78% of the patients die before reaching a hospital.[1] Mortality among admitted patients is 21%.[2] Management of patients who have suffered trauma to the upper airway is always a challenge for the emergency and trauma team. Awareness regarding the possible complications and vigilant monitoring are essential for successful management of these patients. We report the complications encountered while managing a case of tracheal rupture due to a bull horn injury.

## CASE REPORT

A 22-year-old male patient reported to the emergency department with a bull horn injury in the midline of the neck. Head injury and major vascular injury was ruled out. The patient was conscious and distressed but was hemodynamically stable. Local examination revealed a 5–6 cm × 3–4 cm lacerated wound in the lower one-third of the neck in the midline. The trachea was exposed and about three-fourths of the circumference of the third tracheal ring was lost. There was no major bleeding at the site.

The patient underwent emergency primary repair of the wound. He was very distressed and unable to cooperate for awake intubation. The possibility of losing the airway and the anticipated difficulty in bag-mask ventilation following induction with an intravenous induction agent made us opt for inhalational anesthesia. He was therefore induced with halothane and intubated, keeping in readiness all preparations for difficult airway management (i.e., ILMA, lightwand, fiberoptic and finally emergency tracheostomy). Primary repair of the trachea was done over an inserted endotracheal tube.

Postoperatively, elective controlled ventilation (volume-assist/control and pressure limit) was planned for 7–10 days in the ICU. Accordingly, the patient was paralyzed with an intravenous infusion of vecuronium bromide given at the rate of 1 μg/kg/min through a syringe pump. The patient's neuromuscular paralysis was monitored with the train-of-four (TOF) stimulus. Initially the TOF was monitored every 30 min to adjust the dose of vecuronium bromide to obtain a TOF ratio of less than 0.75. Once the dose was adjusted to the required level of neuromuscular blockade, the TOF was monitored every 2 h. Sedation and analgesia was maintained using midazolam and fentanyl infusion as per the protocol followed at our ICU. Midazolam was infused at the rate of 0.03–0.08 mg/kg/h and fentanyl at the rate of 25–50 μg/h. The sedation was titrated between level 5 and 6 (new Sheffield scale). After approximately 8 h of postoperative ventilation, the patient suddenly started coughing and bucking on the endotracheal tube. This was because of the failure of the syringe pump administering the muscle relaxants, which had gone unnoticed. Infusion was restarted after a bolus dose of vecuronium bromide intravenously but, meanwhile, the patient developed subcutaneous emphysema in the neck. The emphysema gradually spread over the chest and upper abdomen and led to decreased compliance of the chest wall, raised peak airway pressure, and caused a fall in the SpO_2_.

**Figure 1:**
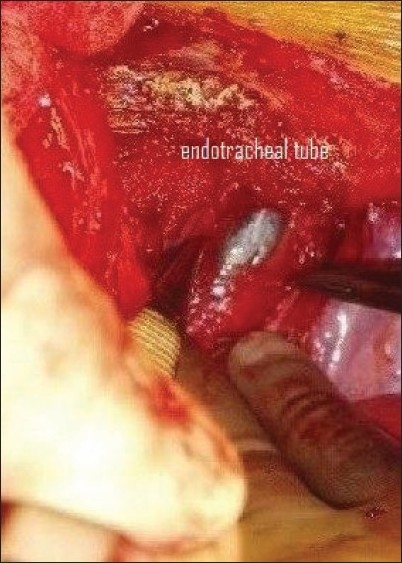
Endotracheal tube visible through slit in the trachea

**Figure 2:**
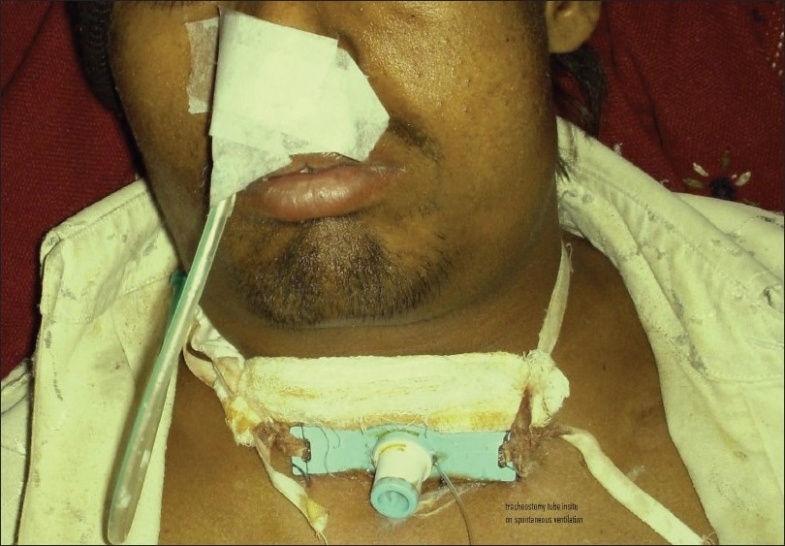
After drainage of surgical emphysema; the patient is on spontaneous ventilation with the tracheostomy tube in situ

The patient was immediately transferred to the operating room. Exploration of the surgical site revealed dehiscence of the tracheal wound [Figure 1], which had led to the subcutaneous emphysema. Repair of the tracheal wound dehiscence was not possible due to both lack of space and lack of tissue for apposition. Additionally, tracheostomy was not possible below the level of the wound because the wound was just above the manubrium sterni. Hence, a tracheostomy tube was inserted through the tracheal wound and the patient was transferred to the ICU for elective controlled ventilation in assist / control volume ventilation mode (rate: 12, TV: 500 ml, PEEP: 5, FiO2: 1, flow rate: 50 l/min, sensitivity 2 l/min). The FiO2 was gradually reduced to 50% over a period of 4–6 h. After approximately 12 h of elective ventilation the neuromuscular blocking drug was stopped. Once the patient started triggering spontaneous breaths, the ventilating mode was changed to synchronized intermittent mandatory ventilation with pressure support (SIMV + PS) (rate: 12, TV: 500 ml, PEEP: 5, FiO2: 0.5, flow rate: 50 l/min, + PS 10. The SIMV rate was gradually reduced to 6. The patient was then shifted to continuous positive airway pressure (CPAP) mode. The rapid shallow breathing index (RSBI) was calculated with a PS of 6 and a PEEP of 5. Once the RSBI was < 100 the patient was disconnected from the ventilator and allowed to breathe spontaneously. Oxygen was administered with the help of a T-piece connection attached to the tracheostomy tube. He was then transferred to the surgical ward on spontaneous ventilation with the tracheostomy tube in situ [Figure 2].

**Figure 3:**
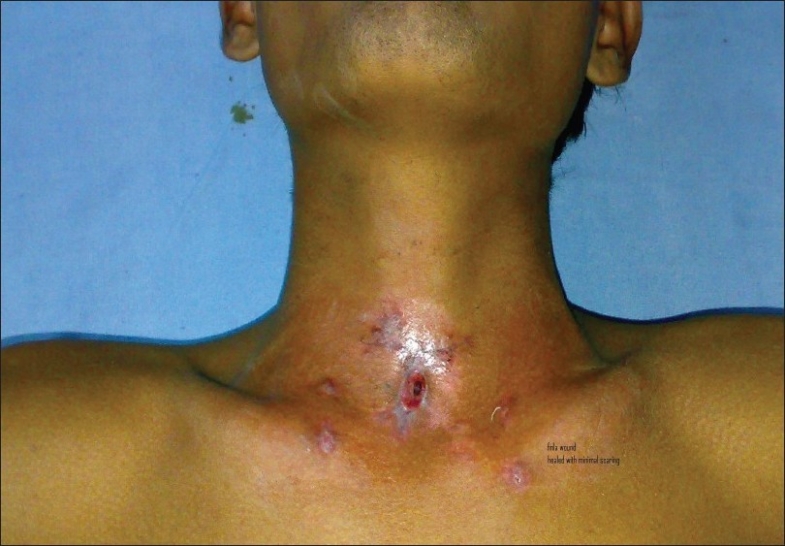
The patient, after removal of the tracheostomy tube

The size of the patient's tracheostomy tube was reduced gradually by the serial exchange method. The wound ultimately healed with minimal scarring [Figure 3].

## DISCUSSION

A standard categorization of penetrating neck injuries by Roon and Christensen[11] divides the neck into three zones. This injury occurred in the lowest zone, i.e., zone 1. The majority of reports in literature suggests primary surgical repair with buttressed suturing as the treatment of choice for tracheal perforation[3–7]; this is to be followed by elective controlled ventilation under deep sedation and muscular paralysis to avoid stress on the suture line and prevent wound dehiscence. Some authors suggest conservative management as an alternative, especially in cases of small lacerations involving the posterior wall only.[8,9] Although one report in literature suggests non invasive positive pressure ventilation (NPPV) as an alternative,[10] all patients ultimately require a period of tracheal intubation and artificial ventilation while the airway heals.

One of the major challenges in managing this patient was in maintaining the airway at the time of induction. In our opinion, rapid-sequence intubation (RSI) would have been deleterious to the patient. The risk of aspiration would have been more since application of cricoid pressure was not feasible in view of the site of the injury. Further, the possibility of losing the airway would be more due to the apnea that follows RSI. In addition, bag-mask ventilation, which might have become necessary if it had turned out to be a difficult airway, would be difficult due to the tracheal rupture. Although clinically the patient did not have signs of a difficult airway, the possibility could not be ruled out because tracheal rupture had resulted in a bloody oral cavity and there was dragging down of the trachea due to loss of the tracheal support provided by the strap muscles of the neck. Hence, we decided to induce anesthesia with an inhalational agent and intubate so to maintain the airway and spontaneous ventilation.

Sedation and analgesia was maintained with a combination of midazolam and fentanyl infusion. The level of sedation was maintained between 5 and 6 of the new Sheffield scale.[12] Sedation levels 5 and 6 are only acceptable in certain cases, e.g., in the patient who is receiving neuromuscular blockers. Heavy sedation is required to ensure the patient's comfort; the aim being to avoid the situation where the patient is chemically paralyzed but not receiving adequate analgesia and sedation.

There is no literature on postoperative subcutaneous emphysema in a case of tracheal injury. Further, we could not locate any protocol for the management of subcutaneous emphysema causing respiratory obstruction. At the time we felt that the best way to manage the case was to relieve the airway obstruction urgently. One challenge was to maintain airway patency after the repeat operation. The only option left to us was to insert a tracheostomy tube at the perforation site.

Following the primary surgical repair, the plan was to electively ventilate the patient for a minimum period of 7–10 days. This would enable the wound to heal without any stress on the suture site. However, during the repeat operation the wound had to be repaired over a tracheostomy tube. The plan then was to wean the patient off the ventilator as early as possible so as to keep him breathing spontaneously and to avoid the possibility of surgical emphysema under positive pressure ventilation. Since the patient required only short-term ventilatory support, no specific weaning protocol was applied. Further, since the patient had a tracheostomy tube in situ, a trial of early disconnection from the ventilator was possible.

The patient was then transferred to the ward on spontaneous ventilation, with his tracheostomy tube in situ. The wound ultimately healed without any complications and with minimal scarring.

## CONCLUSION

Patients with tracheal injury should be electively ventilated until healing of the wound so as to prevent wound dehiscence. In order to avoid complications, such patients should be closely monitored.

Every trauma patient is different. Managing trauma patients needs clinical acumen and intelligence. It calls for team work. Success depends upon the coordination between different specialties. Such cases do not always follow the rule book and, hence, there is always something new to learn and practice while managing them.
